# Problematic Internet Use in Adolescents from Divorced Families: The Role of Family Factors and Adolescents’ Self-Esteem

**DOI:** 10.3390/ijerph18073385

**Published:** 2021-03-25

**Authors:** Rianne van Dijk, Inge E. van der Valk, Helen G. M. Vossen, Susan Branje, Maja Deković

**Affiliations:** 1 Department of Clinical Child & Family Studies, Faculty of Social and Behavioral Sciences, Utrecht University, Heidelberglaan 1, 3584 CS Utrecht, The Netherlands; H.G.M.Vossen@uu.nl (H.G.M.V.); M.Dekovic@uu.nl (M.D.); 2 Department of Youth & Family, Faculty of Social and Behavioral Sciences, Utrecht University, Heidelberglaan 1, 3584 CS Utrecht, The Netherlands; I.E.vanderValk@uu.nl (I.E.v.d.V.); S.Branje@uu.nl (S.B.)

**Keywords:** problematic internet use (PIU), adolescents, divorced families, interparental conflict, triangulation, maternal and paternal warmth, self-esteem

## Abstract

Family functioning is salient in explaining adolescents’ problematic internet use (PIU), and precisely this family functioning is under pressure after parental divorce. Moreover, growing up with divorced parents is identified as a risk factor for PIU. Therefore, examining which factors are associated with adolescents’ PIU after divorce is particularly important. Based on self-report data from *N* = 244 adolescents of divorced families (49.6% boys, *M* = 13.42), structural equation modeling (SEM) was used to examine the associations of PIU with interparental conflict, triangulation, maternal and paternal warmth, and adolescents’ self-esteem. Potential buffering effects of self-esteem were tested, as well as gender differences in associations. The results showed that more triangulation and less maternal warmth were related to higher levels of PIU, but these effects disappeared after adding self-esteem to the models. Adolescent self-esteem did not significantly buffer the effects of the different family factors on PIU, nor were there any significant gender differences in association. Hence, especially adolescents’ self-esteem seems to be a key aspect for PIU in adolescents from divorced families.

## 1. Introduction

Adolescence is a period in which the possibilities of the internet and social media seamlessly align with youngsters’ basic need for more autonomy and increased focus on their peers [1]. Since the current generation of adolescents has grown up with access to computers and the internet from an early age on [2], it is of no surprise that adolescents and young adults have the highest rates of internet use as compared to other age groups [3,4,5]. Although spending moderate amounts of time online can strengthen adolescents’ offline relationships [6,7], excessive internet use is related to mental health problems [8]. This may be attributed to the fact that high-frequent or heavy users are also more likely to be problematic users [9,10]. Problematic internet use (PIU) is not only characterized by heavy use, but by addiction-like behaviors as well [11].

PIU can include different types of online behavior, such as problematic social media use [12,13], online gaming addiction [14], and more general pathological internet use or internet addiction [15]. It involves online behavior that is risky, excessive, or impulsive. As adolescence is a period in which self-control and its underlying fronto-limbic brain circuitry is still developing [16], adolescents are more likely to experience difficulties in regulating their internet use impulses and feel stressed or anxious when they are restricted in their use [15,17]. In particular, this addictive type of online behavior seems harmful for adolescents [18]. For instance, PIU has been related to worse psychosocial functioning [12,18,19,20], and it also appears to be an early sign for other problematic behaviors, such as drug- and alcohol abuse [21]. Given the potential risk of PIU for their development, it is important to identify potential factors associated with adolescents’ PIU.

Along with various other psychosocial and behavioral problems [22,23], PIU is found to be more common in adolescents from divorced families [24,25,26]. Family functioning is thought to be salient in explaining adolescents’ PIU [26,27,28], and precisely this family functioning is under pressure after a divorce or parental separation. That is, not necessarily divorce itself but (deteriorated) family functioning after the divorce is key in explaining adolescents’ post-divorce adjustment [22]. This likely holds for adolescents’ PIU as well, but empirical evidence is lacking thus far. Youngsters are thought to be especially hindered by divorce in case of negative and conflictual family processes [22,29], such as interparental conflict, triangulation, and low parental warmth. Therefore, in the current study, we examined the associations between these family factors and adolescents’ PIU in divorced families. In addition to family factors, self-esteem has consistently been linked to adolescents’ PIU in predominantly non-divorced families [30,31,32], and is considered a fundamental aspect of adolescents’ healthy development [33]. Therefore, we also examined the role of self-esteem and tested if adolescents’ self-esteem could moderate the effects of the family factors on PIU. Lastly, we explored gender differences in associations.

### 1.1. Family Factors and PIU: Interparental Conflict, Triangulation, and Parental Warmth

The assumption that the online world can be used to escape real-life issues and alleviate negative mood has been referred to as compensatory internet use [34]. Such real-life issues often occur in the family context, of which the interparental subsystem is considered most salient [35]. More specifically, interparental conflict has been linked to adolescents’ PIU in (mostly) non-divorced families, both concurrently [36,37,38] and longitudinally [39]. Interparental conflict would prompt adolescents’ escaping behavior into the online world as a way to avoid the stress often accompanying such conflict [40]. Stress has indeed been found to trigger PIU [41], and adolescents who show more cognitive or behavioral strategies to escape from stress are more inclined to engage in PIU [42]. Conflicts during and after the divorce are common [43,44,45], which underscores the need to examine the link between interparental conflict and adolescents’ PIU in divorced families.

When parents actively involve their child in their conflictual interactions, the interparental dynamics that initially have a dyadic character become triadic. Parents can involve children in their disputes by pressuring them to take sides, using them as messengers, or disclosing negative information about the other parent [46,47]. This involvement has been referred to as triangulation and enhances the probability that children experience loyalty conflicts (i.e., feeling caught between parents [48]). Although triangulation is by no means unique to divorced families [49], it is far more likely to occur in the context of divorce [50,51]. Triangulation is considered an important risk for healthy child adjustment after divorce [47,49,52], but its role in adolescents’ post-divorce PIU is unclear. 

In addition to the interparental system and triadic subsystem of both parents and the child, the mother–child and father–child systems are assumed to be vital in adolescents’ post-divorce adjustment [53]. In several studies with Southeast-Asian samples [10,24,54], poor parent–child attachment and a lack of warmth from their parents related to adolescents’ PIU. Similar results were found in adolescents from Spain [55,56,57] and Italy [58]. In a Chinese sample, only the father–child relationship quality was of significance [59], whereas both maternal and paternal warmth were associated with PIU in Greek adolescents [60]. Adolescents may try to compensate for a lack of parental warmth by seeking online support instead [61], increasing the risk that this behavior becomes problematic. Whether interparental conflict, triangulation, and parental warmth are indeed related to adolescents’ PIU after divorce needs to be examined. 

### 1.2. Self-Esteem and PIU

Next to family factors, adolescents’ self-esteem is also thought to be important for PIU, as youngsters become more skilled in reflecting on the self during adolescence [62]. Self-esteem concerns the evaluation of the self and the extent to which people like themselves, and is considered a fundamental aspect of psychosocial functioning [33]. Lower levels of adolescents’ self-esteem are related to higher levels of PIU [30,31,32], as they may try to escape from negative self-evaluation through spending time online and to compensate for their low self-esteem by taking on a different personality and identity on dating-, social networking-, and gaming platforms [32,63]. Adolescents with low self-esteem are also more likely to compare themselves to others online than their peers with a higher self-esteem [63]. Increasingly depending on the online world for confirmation and more positive self-evaluation could then lead to adolescents’ online habits becoming problematic. The tendency to escape from the self has already been linked to other self-destructive behaviors, such as substance abuse [64], and might similarly relate to adolescents’ PIU [14].

In addition to its direct link with PIU, adolescents’ self-esteem could potentially moderate the association between family factors and PIU. It is assumed that adolescents with high self-esteem have better coping resources, protecting them against the adversity of stressful circumstances [33,65], such as negative family factors after divorce. Conversely, those with low self-esteem are thought to be more vulnerable to stressors. One might argue that adolescents who are already more susceptible to showing self-escaping behaviors because of low self-esteem [14], are also more likely to respond with PIU to escape from the stressful circumstances they experience due to post-divorce negative family factors. This seems especially relevant to examine in adolescents from divorced families, who are at risk of reduced self-esteem [66,67,68]. 

### 1.3. Gender and PIU

Adolescents’ gender may also play a role in the associations between family factors and PIU. Based on the idea of gender socialization, a meta-analysis concluded that interparental hostility was more strongly related to emotional reactivity (i.e., anger, distress) and internalizing problems for girls than for boys [69]. Girls’ tendency to place more importance on interpersonal relationships might also make them more sensitive to interparental conflict and triangulation [51,70] and increase their risk of engaging in PIU to escape such stressors. Gender differences in effects of parental warmth on adolescents’ adjustment (i.e., internalizing problems, externalizing problems, and relational aggression) are inconsistent [71,72,73,74]. A stronger link between self-esteem and PIU for boys than girls was found [37], but more research on gender differences in associations between family factors, self-esteem, and PIU is needed.

### 1.4. The Current Study

Opportunities to engage in (problematic) internet behavior increase rapidly in the current digital age. On average, adolescents from divorced families are more at risk for PIU [24,25,26], but research on factors that are associated with this type of problem behavior in adolescents after divorce is lacking thus far. Considering the potential risk of PIU for adolescents’ healthy development [13,21], research on possible associated factors could provide viable information for practitioners working with adolescents from divorced families. In addition, most studies on PIU and related factors are based on Southeast-Asian samples due to the high prevalence rates of PIU in this continent [75,76], but the problem is increasing in Western societies as well [77,78]. Therefore, in the current study we examined PIU and its related factors in Dutch adolescents from divorced families.

More specifically, we first examined if family factors and adolescents’ self-esteem were associated with PIU in adolescents from divorced families. Based on previous research and the idea of compensatory internet use [34], we expected that higher levels of interparental conflict (1a) and triangulation (1b), and less maternal (1c) and paternal warmth (1d), would relate to more PIU. In line with the notion of self-escaping behavior [14,64], we expected lower self-esteem (1e) to be associated with more PIU. Second, we tested whether adolescents’ self-esteem moderated the effect of family factors. Based on the premises of escaping behavior—from the self as well as from stress due to negative family factors—we expected that lower levels of self-esteem would intensify the associations between negative family factors (i.e., more interparental conflict (2a) and triangulation (2b), and lower levels of maternal (2c) and paternal warmth (2d)) and PIU, whereas higher levels of self-esteem would buffer this adversity. Lastly, we explored potential gender differences in the associations between family factors and self-esteem on the one hand, and PIU on the other hand. Based on the idea of gender socialization, we anticipated that interparental conflict (3a) and triangulation (3b) would be more strongly related to PIU in girls than boys. As for maternal warmth (3c), paternal warmth (3d), and self-esteem (3e) we had no specific hypotheses regarding gender differences in the relation to PIU. 

## 2. Materials and Methods

### 2.1. Participants

For the current study, we used data from the 2016 cohort of the cross-sequential research project “Students & Families” [79], which aimed to examine differences in family functioning and child adjustment in divorced versus non-divorced families and whether these differences change over the course of time (i.e., 2006–2018). From a total of 1227 students, we selected adolescents from divorced families, that were in secondary school at the time of the measurement, and that had information on at least 2 of the study concepts. Two adolescents reported having no contact with their mother nor father and were deleted from the sample. This resulted in a final study sample of *N* = 244 adolescents from divorced families.

The study sample consisted of 49.6% boys and 50.4% girls, aged 11 to 17 years, with an average of *M* = 13.42 (*SD* = 1.07). Their level of education varied from low (17.5%), to medium (39.5%), and high (43.0%). On average, parents were divorced or separated *M* = 7.85 years (*SD* = 3.98) at the time of the measurement. Participants’ age at the time of their parents’ divorce ranged from 0 to 16 years, with an average of *M* = 6.22 (*SD* = 3.78). Regarding their living arrangements, 11.6% of the adolescents lived only with their mother, 52.0% reported living mostly with their mother (i.e., minimum of 5 days/nights), 27.6% lived with both parents an equal amount of time (i.e., 3 or 4 days/nights each), and 8.9% reported living mostly or only with their father. 

### 2.2. Measures

#### 2.2.1. PIU

To measure adolescents’ PIU, we used the Problematic and Risky Internet Use Screening Scale (PRIUSS; [80]), that consists of the subscales “social impairment” (6 items), “emotional impairment” (6 items), and “risky/impulsive internet use” (6 items). Adolescents were asked to think of every moment or situation they spent online, irrespective of the device they were using (e.g., computer or mobile phone). Messaging on their mobile phone (i.e., texts) was not included, unless this was through social media platform such as Facebook, WhatsApp or Instagram. Example items of the three scales are: “How often do you fail to create real-life relationships because of the internet?” (social impairment), “How often do you feel irritated when you’re not able to use the internet?” (emotional impairment), and “How often do you lose sleep due to nighttime internet use?” (risky/impulsive). Answers were given on a 5-point Likert scale, ranging from Never (1) to Very often (5). The reliabilities of the scales were good, with Cronbach’s α = 0.79, α = 0.85, and α = 0.85, respectively. 

#### 2.2.2. Interparental Conflict

Interparental conflict was assessed with the “parental conflict” scale (10 items) of the Coparenting Behavior Questionnaire (CBQ; [81]), which measures the level of overt hostile behavior between parents as reported by adolescents. Example items are: “My parents argue with each other”, and “My parents get along well” (reversed). Answers were given on a 5-point Likert scale, ranging from Almost never (1) to Almost always (5). The reliability was considered good, as Cronbach’s alpha was α = 0.91. 

#### 2.2.3. Triangulation

Adolescents’ experience of triangulation was measured with the “triangulation” scale (12 items) of the CBQ [81], that taps into adolescents’ feelings of being caught between parents and getting involved in their conflicts. Example items are: “I feel caught between my parents” and “My mom says bad things about my dad”. Answers given on a 5-point Likert scale, ranging from Almost never (1) to Almost always (5). The scale had a good reliability, as Cronbach’s alpha was α = 0.80.

#### 2.2.4. Maternal and Paternal Warmth

Both maternal and paternal warmth as experienced by the adolescents were measured with 6 items of the “mother’s/father’s warmth” subscale of the CBQ [81]. An example item is: “My mother/father says she/he loves me and gives me hugs”. Answers were given on a 5-point Likert scale, ranging from Almost never (1) to Almost always (5). The reliability was good, as Cronbach’s alpha was α = 0.91 for both maternal and paternal warmth.

#### 2.2.5. Self-Esteem

Adolescents’ self-esteem was measured with the Rosenberg self-esteem scale [82], which taps into global self-worth by measuring both positive (5 items) and negative feelings (5 items) about the self. Example items are: “On the whole, I am satisfied with myself” and “I certainly feel useless at times”. Answers were given on a 5-point Likert scale, ranging from Strongly disagree (1) to Strongly agree (5). The reliability was good, as Cronbach’s α = 0.86.

### 2.3. Procedure

Participants of the 2016 cohort of the “Students & Families” research project were recruited at 17 different Dutch schools throughout the Netherlands. Schools were contacted by graduate students of the Faculty of Social Sciences at Utrecht University. After a school was informed about the research and had given its consent, adolescents’ parents were informed of the study through a letter and could indicate if they did not want their adolescent to participate in the study (i.e., passive consent), but none of the parents did. The adolescents themselves filled out the questionnaires individually and anonymously. Questionnaires were introduced, explained and administered in class during school hours. Students could withdraw their participation at any time while filling out the questionnaire, and without giving any reason. 

### 2.4. Statistical Analyses

We first conducted (latent) confirmatory factor analyses (CFAs) for all study variables using structural equation modeling (SEM), as latent constructs are preferred over the use of scaling scores (i.e., mean- or sum scores; [83]). We then saved the individual factor scores of our latent measurement models for subsequent analyses. This is opposed to using a simultaneous approach (i.e., modeling the measurement part and structural part simultaneously), in order to circumvent power issues, to prevent interpretational confounding, and to avert misinterpretation due to potential misspecification in the measurement models permeating into the structural model [83]. Hence, the saved factor scores were used as observed scores the regression models. The diagonally weighted least squares (DWLS)-estimator was used for all CFAs, as this estimator provides less biased factor loadings for Likert-type data when compared to a (robust) maximum likelihood estimator (ML or MLR; [84]). Goodness-of-fit statistics were used to evaluate the model fit, for which the following cutoffs were used: Comparative Fit Index (CFI) > 0.95, Root Mean Square Error of Approximation (RMSEA) < 0.06, and Standardized Root Mean Square Residual (SRMR) < 0.08 [85].

After constructing the variables, we used SEM for regressing PIU on interparental conflict, triangulation, maternal and paternal warmth, and self-esteem. As most of the variables showed univariate non-normality (which is to be expected and common in psychological research [86]), percentile bootstrapping was used to estimate the regression estimates, which is preferred over other methods [87,88]. A full information likelihood estimator was used to account for missing data. In the regression analyses, the variables were added to the models in a stepwise manner: First, the main effects of family factors and gender were entered in the model simultaneously. Second, the main effect of self-esteem was added to the model. Third, we checked for gender differences in associations between the family factors, self-esteem, and PIU by entering interaction terms one-by-one (i.e., four different family factors * gender and self-esteem * gender). Fourth, interactions terms were again entered one-by-one to examine a potential buffering effect of self-esteem (i.e., four different family factors * self-esteem). All analyses were performed in the statistical software program “R” version 3.6.3 [89], using the “Lavaan” package [90].

## 3. Results

### 3.1. Confirmatory Factor Analyses

The study variables were examined using CFAs. In Table 1, the model fit indices and range of factor loadings are presented for each of the constructs. As PIU is thought to underly the three PIU factors (i.e., social impairment, emotional impairment, and impulsive/risky internet use) that were measured with multiple items per scale, a second-order CFA was used. The CFAs for PIU, interparental conflict, maternal warmth, and paternal warmth all had a good model fit, whereas the model fit for the CFAs of triangulation and self-esteem were not adequate at first. Hence, after inspecting the modification indices for the triangulation construct, we correlated the error terms of the items that were formulated exactly the same, but for mother and father separately (e.g., “my mom says negative things about my dad” and “my dad says negative things about my mom”). For self-esteem, we modeled a second-order factor, accounting for method variance (i.e., five positively and five negatively formulated questions). This resulted in a good model fit for all constructs, of which the individual factor scores were saved for the regression models. 

### 3.2. Descriptive Statistics

In Table 2, the descriptive statistics of all study variables are presented. Higher levels of triangulation, less maternal warmth, and lower levels of self-esteem were significantly correlated with higher PIU, whereas interparental conflict and paternal warmth were not significantly associated with PIU. Interparental conflict and triangulation strongly correlated and were both negatively associated with maternal warmth and adolescents’ self-esteem. Interparental conflict was also negatively associated with paternal warmth, in contrast to triangulation that did not correlate with paternal warmth. The strengths of the correlations varied from small to moderate. 

### 3.3. Regression Models

As interparental conflict and triangulation were highly correlated (*r* = 0.75) but seemed to have unique associations with some of the other constructs (see Table 2), we decided to run the regression analyses for interparental conflict and triangulation separately. The estimates of the multiple regression models are depicted in Table 3. These results show that, in the conflict model, 6% of PIU was explained by the family factors. In this model, maternal warmth was negatively associated with PIU (i.e., *p* = 0.052, but 95% CI of the 10,000 bootstraps did not include 0, as [−0.17, −0.003]) when taking into account gender, interparental conflict, and paternal warmth. That is, less maternal warmth was related to higher levels of PIU in adolescents. However, none of the other factors in this model were significant. 

In the triangulation model, 10% of the variance in PIU was explained by the family factors. More specifically, triangulation was significantly associated with PIU, after taking into account gender, maternal warmth, and paternal warmth. Hence, more triangulation was related to higher levels of PIU in adolescents. None of the other factors in this model were significant. Furthermore, when adding self-esteem to both the interparental conflict and triangulation models, all other significant associations disappeared, and the explained variance increased to 13% (ΔR^2^ = 0.07) and 15% (ΔR^2^ = 0.05) for the interparental conflict and triangulation model, respectively. Lastly, none of the interaction terms were significant, indicating that associations did not differ based on gender, neither did self-esteem moderate the effect of interparental conflict, triangulation, or maternal and paternal warmth.

## 4. Discussion

The overarching aim of the current study was to gain more understanding of adolescents’ PIU in divorced families. Growing up after a parental divorce seems to be a risk for PIU in adolescents [24,25,26], yet research on PIU after divorce has been lacking thus far. Since family functioning is reported to be essential in adolescents’ PIU [26,27], and it is precisely this family functioning that is under pressure after divorce, identifying factors that are associated with adolescents’ post-divorce PIU is crucial. Hence, our first goal was to examine if family factors (i.e., interparental conflict, triangulation, and parental warmth) and self-esteem were related to adolescents’ PIU after divorce. Moreover, through moderation analyses, possible factors that either exacerbate or buffer the adversity of negative family factors were examined. Therefore, the second goal was to test whether adolescents’ self-esteem would moderate the associations between family factors and PIU. Lastly, potential gender differences in the associations were explored.

### 4.1. Family Factors and PIU

Based on our results, we only found partial evidence for our first research question. More specifically, when considering the models with family factors only (i.e., model 1), higher levels of triangulation (1b) and less maternal warmth (1c; i.e., only in the interparental conflict model, not the triangulation model) were associated with more PIU, but interparental conflict (1a) and paternal warmth were not (1d). This latter finding could be due to the fact that after divorce, children on average spend a larger amount of time with their mother compared to their father [91]—which is also true in the current sample—and, therefore, maternal warmth may have more impact on children than paternal warmth. This may also explain why our findings differ from previous studies, in which significant associations were found between paternal warmth specifically and PIU [59,60], as those samples involved predominantly non-divorced families. Additionally, unlike these other studies, we entered multiple family factors in the models at once, automatically controlling for interparental conflict/triangulation. 

As for interparental conflict and triangulation, despite the high correlation between the two constructs (*r* = 0.75), our results suggest that merely witnessing interparental conflict was not associated with PIU, which is in contrast with previous studies on this association [36,39]. Yet, parents’ behaviors that actively involve adolescents in disputes with their ex-spouse, i.e., triangulation, do seem to affect adolescents, which aligns with the notion that feeling caught between parents after divorce is particularly stressful for adolescents [49,52], and is related to escaping into the online world to avoid stress and alleviate negative mood as proposed in the compensatory internet use view [34]. These intentions to escape from stress and alleviate negative mood should be examined more thoroughly as potential underlying mechanisms of the link between triangulation and PIU.

### 4.2. Self-Esteem and PIU

Despite the potential impact of triangulation and maternal warmth, further analyses (i.e., model 3) revealed that especially adolescents’ self-esteem was key in explaining PIU, when compared to the family factors. That is, the associations between PIU and the family factors (i.e., triangulation and maternal warmth) were no longer significant after adding self-esteem to the model. Consistent with our expectations, lower self-esteem was related to more PIU in adolescents (1e). This finding is in line with the notion that the tendency to escape from the self is linked to self-destructive behaviors [64]. Adolescents are thought to escape from negative self-evaluation through taking on a different online personality and identity [32,63], but they also compare themselves to others online more [63]. When the dependency on the online world increases, the risk for PIU does as well. It seems as if the need to escape the self is more strongly related to adolescents’ PIU after divorce than escaping stress accompanied with negative family factors. This is specifically a topic of concern for youngsters from divorced families, as self-esteem is one of the aspects of adolescents’ adjustment most consistently reported as affected by a parental divorce [68].

Our hypotheses that self-esteem would moderate the associations between the (negative) family factors and PIU (2a–d) were not supported by our findings. We anticipated that the tendency to escape from the self [14,64], due to low self-esteem would exacerbate the tendency to escape from negative real-life issues (i.e., double risk), whereas high levels of self-esteem could buffer negative family factors (i.e., better coping resources; [65]). However, no such moderation effects were found. When inspecting Table 2 more closely, the family factors were moderately correlated with adolescents’ self-esteem. In addition, the effects of triangulation and maternal warmth disappeared when adding self-esteem to our regression models (i.e., third step in Baron and Kenny’s mediation method; [92]), suggesting that self-esteem might not necessarily buffer family adversity after divorce but rather act as an underlying mechanism in the association between adverse family factors and PIU. Self-esteem has indeed been suggested to mediate the link between family functioning and PIU [93], interparental conflict and PIU [37], and parental warmth and PIU [94]. Unfortunately, congruent with our study, all these studies involved cross-sectional data. Although our results also hint towards self-esteem as an underlying mechanism in the relation between family factors and PIU, longitudinal data are required to test such mediation hypotheses due to the assumption of time precedence [95]. The cross-sectional nature of our data also prohibits from drawing conclusions regarding causality and bidirectionality. For instance, in another study, deterioration of family function was found after internet addiction initiation of youngsters [39]. More longitudinal research to examine such processes is necessary.

### 4.3. Gender and PIU

Finally, we examined potential gender differences in associations between the family factors (3a–d), self-esteem (3e), and PIU. Again, no significant moderation effects were found, despite the gender differences in correlations, for example, between interparental conflict and PIU (i.e., *r* = -.02 for boys and *r* = 0.21 for girls). Yet, our sample size may have been inadequate to detect small effects of the interaction terms (i.e., Δ*R*^2^ ≥ 0.01) in the regression models, as our post-hoc power for these moderation effects given the correlations and the effect sizes ranged from 0.30 to 0.50 [96,97]. In addition, we may have found more prominent gender differences if we used a more specific PIU measure. That is, boys are more inclined to develop an internet addiction than girls who are more vulnerable for problematic social media use [98]. Lastly, the developmental stage (e.g., early, mid-, and late adolescence) may play a role in associations as well. Future research could benefit from making distinctions in PIU types, as well as examining PIU as a broad construct, and differentiating between developmental stages during adolescence.

### 4.4. Limitations and Strengths

In addition to the limitations of sample size and cross-sectional character of the data, it should first be noted that all information is based on self-report data of adolescents. On the one hand, this offers a unique insight into the perceptions of adolescents themselves, but on the other hand, the views of other family members are missing. Second, although examining PIU in non-Southeast-Asian countries is important, and we therefore used a Dutch sample of adolescents, the prevalence of PIU is lowest in the Netherlands when compared to other European countries [12], which could have caused less variance in PIU compared to previous studies with samples from different countries. Third, the conclusions of the current study should be interpreted cautiously, as the associations we found were mostly of small effect size. Lastly, since our focus was on divorced families, the time spent with parents might contribute to the associations between family factors and adolescents’ PIU, as, for instance, vulnerability to interparental conflict is found to depend on contact with father [99]. Incorporating such aspects of divorced families is important for future research with larger sample sizes but was beyond the scope of the current study. 

Despite these limitations, the current study was one of the first to examine PIU of adolescents growing up in a family after divorce. A large number of studies has been carried out on the potential negative consequences of parental divorce [53,68], but contemporary problems among adolescents after divorce, such as PIU, have been investigated to a much lesser extent. Moreover, the present study goes beyond that, by also taking into account several factors of different family subsystems in examining post-divorce PIU. The use of factor loadings for constructing the variables and bootstrapping methods for obtaining the regression estimates add to the strength of the study.

## 5. Conclusions

The current study guides future research by offering a first step in unravelling family and intrapersonal factors associated with adolescents’ PIU after divorce. Our findings are partly in line with the idea of compensatory internet use, as more triangulation and less maternal warmth increase the risk for adolescents’ PIU after divorce. Moreover, adolescents’ self-esteem in particular was key in explaining post-divorce PIU. This underscores the notion that the tendency to escape from the self because of negative self-evaluation, is linked to self-destructive behaviors of adolescents. This is specifically a topic of concern for youngsters from divorced families, as self-esteem is one of the aspects of adolescents’ adjustment most consistently reported to be affected by a parental divorce. Future research should examine the interplay of (negative) family factors and self-esteem after divorce in predicting PIU more thoroughly and over time, and test self-esteem as a potential mediating mechanism underlying the link between family factors and PIU after divorce. When more research is conducted on this topic, practitioners who work with adolescents from divorced families and their parents can be informed on the issue of adolescents’ PIU after divorce, related (family) factors, and working mechanisms.

## Figures and Tables

**Table 1:** Model fit statistics and factor loadings for the different confirmatory factor analyses (CFAs).

	χ^2^ (df)	p	CFI	RMSEA	SRMR	Factor Loadings
IPC	56.51 (35)	0.012	0.989	0.052	0.074	[0.58–0.81]
Triangulation *	65.13 (50)	0.074	0.981	0.039	0.073	[0.20–0.73]
Maternal warmth	2.42 (9)	0.983	1.000	0.000	0.036	[0.73–0.85]
Paternal warmth	0.99 (9)	0.999	1.000	0.000	0.02	[0.70–0.85]
Self-esteem **	34.09 (33)	0.415	0.999	0.012	0.06	[0.69–0.82]
Positive items						[0.55–0.84]
Negative items						[0.68–0.80]
PIU	95.29 (132)	0.993	1.000	0.000	0.074	[0.79–0.98]
PIU social						[0.43–0.72]
PIU emotional						[0.65–0.75]
PIU impulsive						[0.63–0.75]

* Model fit indices after correlating the error terms of similarly formulated items. ** Model fit indices after controlling for method variance. Notes. A DWLS estimator and 10,000 bootstrap draws were used for all CFAs. IPC = interparental conflict; PIU = problematic internet use.

**Table 2:** Correlations, means, and standard deviations of the study variables.

	1.	2.	3.	4.	5.	6.
1. Interparental conflict	-					
2. Triangulation	0.75 ***	-				
3. Maternal warmth	−0.29 ***	−0.35 ***	-			
4. Paternal warmth	−0.18 **	−0.10	0.19 **	-		
5. Self-esteem	−0.30 ***	−0.44 ***	0.34 ***	0.25 ***	-	
6. PIU	0.13	0.28 ***	−0.21 **	0.02	−0.34 ***	-
M	2.08	1.90	4.20	3.85	3.53	1.77
SD	0.89	0.67	0.87	1.12	0.62	0.67

*** *p* < 0.001 ** *p* < 0.01. Notes. The means and SDs are based on mean scores of the scales, for the correlations and further analyses we used the saved (latent) factor scores. PIU = problematic internet use.

**Table 3:** Regression estimates for PIU: interparental conflict and triangulation models.

	b	SE b	β	p	95% CI	R^2^
1. Model 1 (family factors)						
IPC/Triangulation	0.04/**0.29**	0.04/**0.12**	0.09/**0.22**	0.261/**0.012**	[−0.03, 0.11]/**[0.08, 0.53]**	0.06/0.10
Warmth Mother	**−0.08**/−0.06	**0.04**/0.04	**−0.19**/−0.15	**0.052**/0.135	**[−0.17, −0.00]**/[−0.15, 0.02]	
Warmth Father	0.03/0.01	0.03/0.03	0.07/0.03	0.334/0.714	[−0.03, 0.08]/[−0.05, 0.07]	
Gender	0.03/0.04	0.05/0.05	0.05/0.06	0.504/0.409	[−0.07, 0.13]/[−0.06, 0.14]	
2. Model 1 + self-esteem						
IPC/Triangulation	0.01/0.13	04/0.13	0.03/0.10	0.721/0.317	[−0.05, 0.09]/[−0.12, 0.39]	0.13/0.15
Warmth Mother	−0.04/−0.04	0.04/0.04	−0.08/−0.08	0.375/0.390	[−0.12, 0.04]/[−0.12, 0.04]	
Warmth Father	0.04/0.03	0.03/0.03	0.10/0.06	0.126/0.408	[−0.01, 0.10]/[−0.04, 0.10]	
Gender	−0.01/0.01	0.05/0.05	−0.02/0.01	0.775/0.897	[−0.12, 0.08]/[−0.09, 0.11]	
Self-esteem	**−0.29/−0.26**	**0.07/0.08**	**−0.33/−0.31**	**0.000/0.001**	**[−0.43, −0.16]/[−0.42, −0.11]**	
3. Model 2 + interactions gender						
IPC/Tria * Gender	0.08/0.23	0.06/0.21	0.15/0.14	0.212/0.277	[−0.04, 0.21]/[−0.17, 0.65]	0.14/0.16
Warmth M * Gender	−0.05/−0.09	0.08/0.08	−0.08/−0.17	0.528/0.228	[−0.19, 0.11]/[−0.23, 0.07]	0.13/0.16
Warmth F * Gender	0.04/0.04	0.06/0.06	0.07/0.06	0.490/0.560	[−0.08, 0.16]/[−0.09, 0.16]	0.13/0.16
Self-esteem * Gender	0.02/−0.08	0.13/0.14	0.02/−0.07	0.881/0.552	[−0.23, 0.28]/[−0.35, 0.19]	0.13/0.16
4. Model 2 + interactions SE						
IPC/Tria * Self-esteem	0.05/0.07	0.10/0.29	0.06/0.03	0.586/0.820	[−0.15, 0.23]/[−0.50, 0.65]	0.13/0.15
Warmth M * Self-esteem	0.06/0.09	0.12/0.10	0.06/0.09	0.612/0.405	[−0.21, 0.24]/[−0.15, 0.25]	0.13/0.16
Warmth F * Self-esteem	−0.09/−0.10	0.07/0.09	−0.10/−0.11	0.207/0.267	[−0.20, 0.08]/[−0.22, 0.13]	0.14/0.17

Notes. Estimates before the slash are for the interparental conflict models, after the slash are the estimates for the triangulation models. A number of 10,000 bootstrap draws were used for all models. The printed estimates of the main effects are based on models with the main effects only (i.e., no interaction effects included). The interaction terms are tested one-by-one. Estimates presented in bold refer to statistically significant estimates. IPC = interparental conflict; Tria = triangulation; Warmth M = maternal warmth; Warmth F = paternal warmth; SE = self-esteem. * it denotes an interaction term.
